# A platform for high-throughput bioenergy production phenotype characterization in single cells

**DOI:** 10.1038/srep45399

**Published:** 2017-03-28

**Authors:** Laimonas Kelbauskas, Honor Glenn, Clifford Anderson, Jacob Messner, Kristen B. Lee, Ganquan Song, Jeff Houkal, Fengyu Su, Liqiang Zhang, Yanqing Tian, Hong Wang, Kimberly Bussey, Roger H. Johnson, Deirdre R. Meldrum

**Affiliations:** 1 Center for Biosignatures Discovery Automation, The Biodesign Institute, Arizona State University, 1001S. McAllister Ave., Tempe, AZ 85287, USA

## Abstract

Driven by an increasing number of studies demonstrating its relevance to a broad variety of disease states, the bioenergy production phenotype has been widely characterized at the bulk sample level. Its cell-to-cell variability, a key player associated with cancer cell survival and recurrence, however, remains poorly understood due to ensemble averaging of the current approaches. We present a technology platform for performing oxygen consumption and extracellular acidification measurements of several hundreds to 1,000 individual cells per assay, while offering simultaneous analysis of cellular communication effects on the energy production phenotype. The platform comprises two major components: a tandem optical sensor for combined oxygen and pH detection, and a microwell device for isolation and analysis of single and few cells in hermetically sealed sub-nanoliter chambers. Our approach revealed subpopulations of cells with aberrant energy production profiles and enables determination of cellular response variability to electron transfer chain inhibitors and ion uncouplers.

Cellular heterogeneity at the functional and biomolecular level plays a central role in normal and disease states *in vivo*. Increasing experimental evidence supports the notion of cell-to-cell variability as one of the key determinants in carcinogenesis and tumor progression in the context of clonal evolution mediated by complex interactions of cancer cells with their microenvironment^1^,^2^,^3^,^4^. The bioenergy production phenotype of cells can be reprogrammed in response to a variety of stimuli and perturbations^5^. Dysfunction of mitochondria, which produce bioenergy in form of adenosine triphosphate (ATP) through oxidative phosphorylation (OXPHOS), has been associated with a variety of neurodegenerative diseases, including Alzheimer’s^6^,^7^ and Parkinson’s^8^. Similarly, alteration in energy metabolism manifested as an upregulation of oxidative glycolysis in cancer cells (Warburg effect) has been recognized as one of the hallmarks of cancer^9^. The continuous research in this field continues to reveal new insight into the complexity of energy production phenotypes in tumors and their microenvironment^10^. It is conceivable that changes in cellular energy production may be used as a biosignature to detect changes in cellular states^11^,^12^, e.g. from a normal to a pre-malignant to a metastatic state. However, intrinsic cellular heterogeneity in the energy production profile necessitates studies capable of resolving its characteristics with single cell resolution^13^. Ensemble averaged approaches based on the use of 10^3^–10^7^ cells obscure contributions from individual cells or small subpopulations with abnormal phenotypes that may be the drivers of population survival and proliferation after treatment^1^,^14^.

Spurred by the growing interest in studying energy metabolism at the single cell level, several technologies have been developed to address this need. Oxygen consumption and extracellular acidification (pH) by cells are important indicators of metabolic activity and can serve as proxies for measuring the balance between OXPHOS and glycolysis. While several commercially available platforms for measuring oxygen consumption rate (OCR) in bulk samples based on electrochemical^15^,^16^,^17^ or optical^18^,^19^ sensors exist, only the technology developed by Seahorse (Agilent Technologies, Santa Clara, CA) enables measurements of both OCR and extracellular acidification rate (ECAR). Underscoring the importance of bioenergy metabolism profiling are 2,231 published OCR/ECAR bulk cell studies performed since 2009 with the Seahorse platform alone. However, none of these technologies offer the sensitivity necessary to perform measurements at the single cell level. An experimental platform based on optical sensing of oxygen in hermetically sealed microchambers containing single cells has been developed and optimized earlier by our group specifically for OCR characterization in individual cells^20^,^21^,^22^,^23^. A conceptually similar approach has been demonstrated recently to perform OCR measurements in individual mitochondria^24^. Despite the capability to perform measurements at the single- cell or single-mitochondrion level, the applicability of two methods in biomedical research is limited by low throughput and single-parameter (OCR) readout.

We report on an integrated platform – the “Cellarium” – that enables combined characterization of OCR and ECAR of single cells with a throughput of up to 1,000 individual cells per assay. The measurements are based on ratiometric optical sensing of oxygen and protons in hermetically sealed microwells. Oxygen concentration and pH in the microwells are measured in real time as alterations in the emission intensity of the corresponding thin-film extracellular sensors. An additional fluorophore is incorporated into the thin-film as a reference that is inert to changes in oxygen concentration and pH. Technical characteristics of the platform, implementation details and experimental validation are presented. We found marked heterogeneity in cellular energy production phenotype under normal growth conditions and in response to perturbations of the mitochondrial electron transport chain (ETC). Our data revealed the existence of subpopulations of cells with both low OCR and ECAR under control conditions and in response to ETC inhibitors and proton uncouplers.

Compared to other platforms, the Cellarium enables simultaneous measurements of OCR and ECAR with single cell resolution with markedly higher throughput. As such, our method may be utilized for studying shifts in the bioenergy production balance between OXPHOS and glycolysis—a feature occurring frequently in cancer initiation and progression—in the context of cellular heterogeneity and detection of rare cells with distinctive behavior.

## Results

### Device design and operation

**Figure 1:**
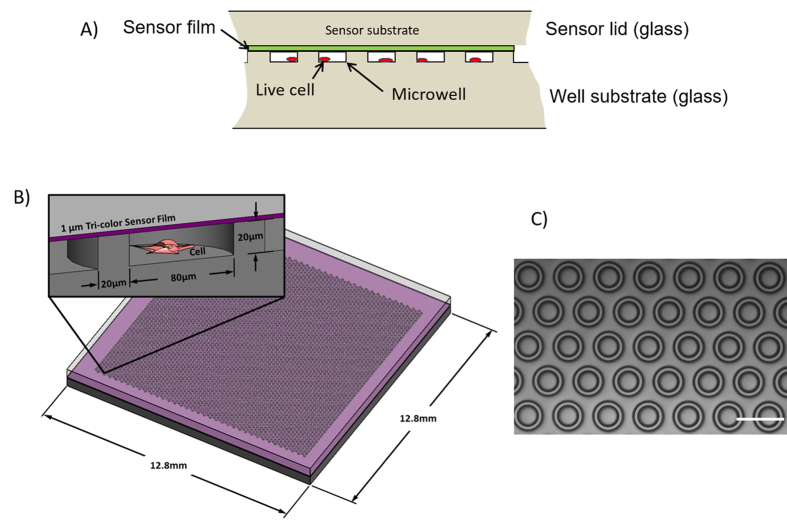
Conceptual approach and device design of the Cellarium technology. (**A**) Single cells are enclosed in hermetically sealed microchambers that contain an extracellular thin-film optical sensor. The sensor emission intensity changes in real time in response to alterations in analyte concentration inside the chamber; (**B**) A microwell array of 4,096 microwells used in the study. The arrays are microfabricated in glass and are optically transparent for readout; (**C**) A transmission light micrograph of a region of the microwell array. Scale bar: 200 μm.

**Figure 2:**
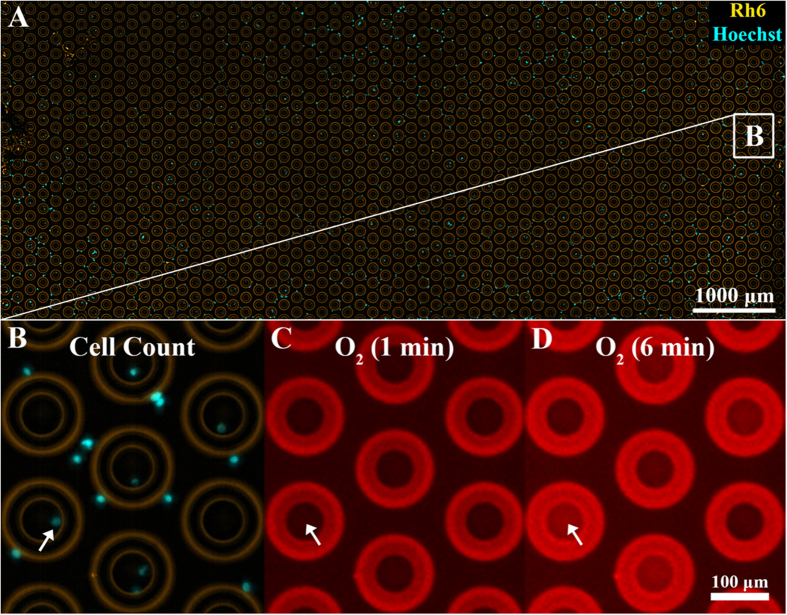
The Cellarium data collection. (**A**) Overlay of micrographs taken in the rhodamine (Rh6) and Hoechst spectral channels of an array of 1,033 microwells (yellow) after random seeding of cells stained with the Hoechst 33342 nuclear DNA marker (blue); (**B**) Zoom-in of the area indicated in (**A**). The light blue spots represent the stained cell nuclei. Cell seeding conditions were optimized to maximize single cell occupancy of the wells (arrow); (**C**) Micrograph of the same area as in (**B**) taken in the oxygen sensor channel at the beginning of an assay. The outside and inside of the wells show the same sensor intensity; (**D**) The same region of the array as in (**C**) at 6 minutes after assay start. As a result of cellular respiration, the emission intensity of the oxygen sensor inside, but not outside, of the sealed wells increases. The respiration rate is determined by calculating the slope of the sensor intensity response as a function of time.

The core Cellarium platform consists of an array of microwells of 64.5 pL volume that can be randomly seeded with cells, hermetically sealed with a sensor lid, and interrogated by fluorescence microscopy over time (Fig. 1A). The microwells with 80 μm diameter and 20 μm depth are arranged as a 64 × 64 array of wells (Fig. 1B,C). The sensor lid contains a plain substrate coated with a thin polymer film containing fluorescent sensors for detecting the change in oxygen concentration and pH (Supplementary Figure 1A). As cells consume oxygen and acidify the interior of the wells, the sensors respond to the changing concentrations by changing their emission intensity (Fig. 2, Supplementary Figure 1B–E). Hermetically sealed microwells are produced by bringing together the microwells and the sensor lid inside of a custom fixture that provides a controlled amount of pressure necessary for sealing (Supplementary Figure 2). Once cells have consumed all oxygen in the well, the well seal is tested by purging the test chamber with oxygen and monitoring sensor response for an additional period of time. The final time point includes a cell count image at high resolution to allow well responses to be normalized by cell number. Extracted sensor intensity for each microwell is analyzed as a function of time to determine OCR and ECAR per well that is then normalized to the number of cells within the well (Supplementary Figure 3).

### Hermetic seal validation

Hermetic sealing of the microwells was tested by flowing 100% oxygen above the medium to saturate the concentration of dissolved oxygen in the fixture. The presence of a hermetic seal of the microwells was confirmed if the oxygen sensor inside the microwells did not respond to the increase in dissolved oxygen concentration on the outside. Using this technique we determined an average number of sealed wells per assay to be 70%. Microwells that were determined as leaky were discarded from further analysis.

### Combined measurements of basal OCR and pH of normal mammary epithelial and metastatic breast cancer cells

**Figure 3:**
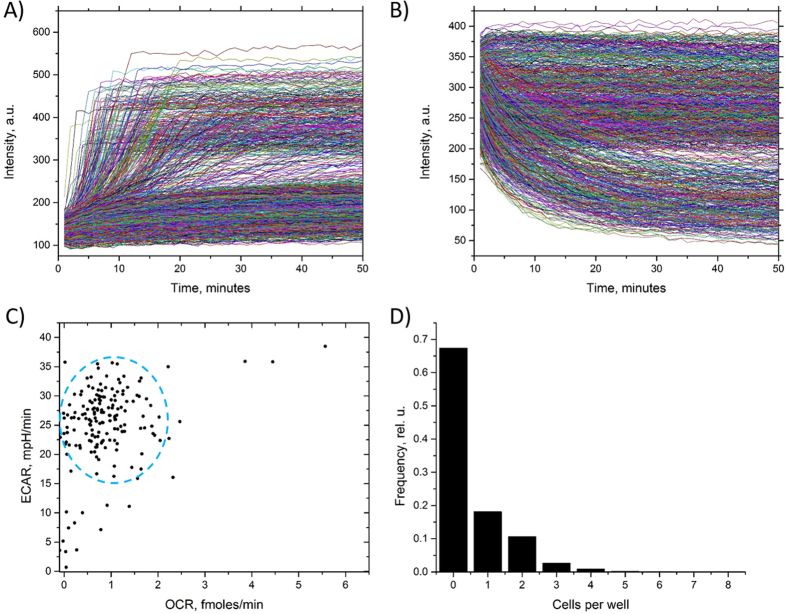
Representative data profiles produced by Cellarium during a typical assay. Each curve in (**A** and **B**) represents the temporal profile of oxygen and pH sensor response, correspondingly, of one individual well of the array. (**A**) Oxygen sensor response. A characteristic response following the Stern-Volmer kinetics of sensor quenching by oxygen is observed in responding wells. The non-responding wells showed mostly a flat response with a slight increase in intensity during the entire experiment; (**B**) pH sensor response. The pH sensor intensity in responding wells decreased 2–3-fold as a result of extracellular acidification in the microwell; (**C**) A combined representation of OCR and ECAR calculated from the data shown in (**A** and **B**). In addition to the main cluster (blue dashed oval), several cells with high or low both OCR and ECAR are present, while the majority of cells exhibit a highly variable profile; (**D**) Measured cell occupancy in the wells. The cell seeding conditions were optimized to maximize the number of wells with single cell occupancy, which resulted in a significant portion of empty wells.

To determine the utility of the method to study cellular energy production heterogeneity we conducted a series of experiments where we measured OCR and ECAR of two human cell types with previously studied energy production profiles. We used human metastatic breast cancer (MDA-MB-231) cells that were demonstrated to have low OCR/ECAR ratio^25^. In addition, we used human mammary epithelial (HME1) cells that were shown to have a lower ECAR than MDA-MB-231 cells^26^. After cell seeding onto the microwell array (Fig. 2A,B), oxygen consumption and extracellular pH data were collected by measuring changes in the emission intensity of the corresponding sensors as a function of time (Fig. 2C,D). The emission intensities of the oxygen and pH sensors of a typical assay with MDA-MB-231 cells are shown in Fig. 3A and B, respectively. The data in the graph represent the entire assay consisting of a portion of the 4,096 well array (1,033 wells) with each curve representing the sensor response from one well. There were 342 wells (33%) occupied by 1 or more cells (the rest of the wells were empty) with 190 wells (18%) containing one cell. Out of 190 single-occupancy wells, 176 wells exhibited a pronounced response (Fig. 3C). Over more than 50 assays, we achieved an average single cell occupancy of 17%. The oxygen sensor intensity in the occupied wells increased due to cellular oxygen consumption and exhibited a typical Stern-Volmer kinetic characteristic of dynamic quenching by oxygen (Online Methods, Fig. 3A, Supplementary Figure 1C). The response terminated with a plateau indicating that the entire amount of dissolved oxygen in the microwell was consumed by the cell(s). On average, the oxygen sensor intensity increased 2.7 fold from the beginning of the assay to the plateau phase, corresponding to the typical sensor response obtained during calibration (Supplementary Figure 1B and C). The empty wells showed a radically different, mostly flat, sensor response with only a slight increase in intensity. The slight increase was found to be caused by oxygen consumption by the oxygen sensor via singlet oxygen generation as a result of sensor excitation. The seal test run at the end of the assay showed that all wells with single cell occupancy were hermetically sealed.

**Figure 4:**
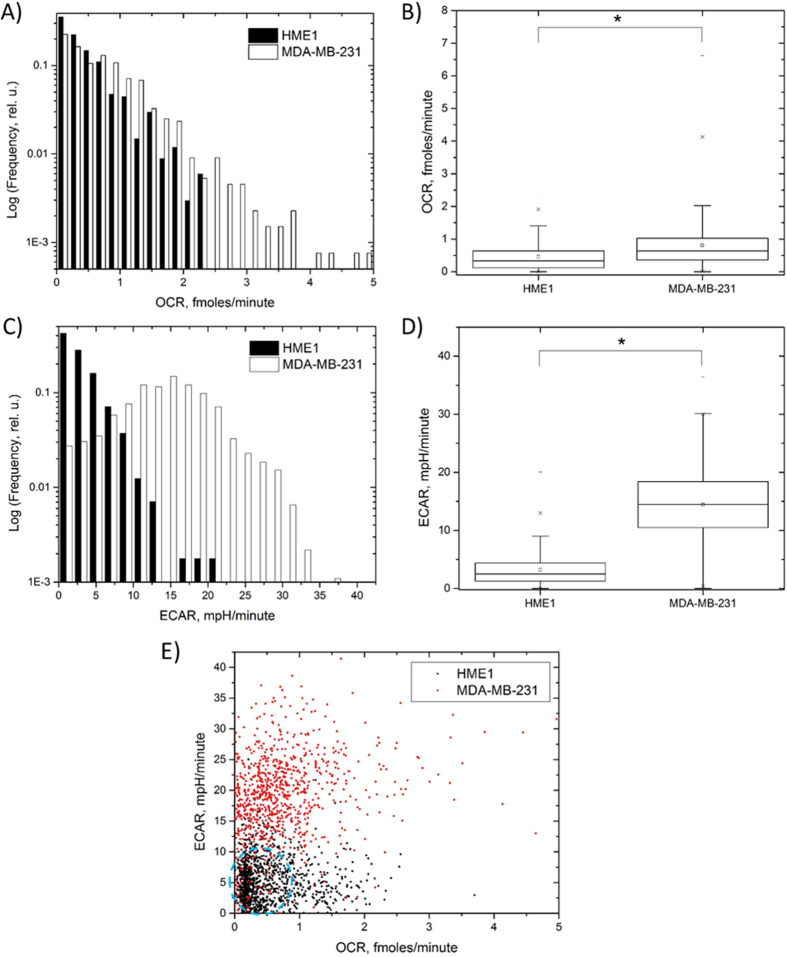
OCR and ECAR comparison of HME1 and MDA-MB-231 cells. (**A**) OCR single cell distribution shows a higher relative fraction of cells with OCR 0.8–4.8 fmoles/min with a lower fraction in the range of 0.2–0.6 fmoles/min in MDA-MB-231 cells; (**B**) Direct comparison of OCR values between HME and MDA-MB-231 cells shows a significant (p = 8.7·10^−19^, KS test) difference. The difference is more pronounced in the ECAR values as evidenced by the presence of larger ECAR values in MDA-MB-231 cells (**C**) and direct comparison of distribution parameters (**D**). (**E**) Comparison of combined OCR and ECAR between the two cell lines. While the majority of MDA-MB-231 cells show markedly higher ECAR values, a fraction of cells overlap with HME1 profile (blue dashed oval).

Contrary to the oxygen sensor response, the pH sensor intensity is higher at high pH (Supplementary Figure 1D, E). Therefore, the sensor intensity is seen to decrease in the wells containing one or more cells due to the pH lowering, whereas sensor intensity in empty wells remained flat (Fig. 3B). The overall distribution of the oxygen and pH sensor response showed distinct well-to-well differences in slopes, with wells containing larger numbers of cells depleting oxygen and lowering pH markedly faster than those with only a few or one cell. Furthermore, the sensor response kinetics in the wells containing one cell also varied significantly from well-to-well reflecting the overall heterogeneity in cell energy production phenotype of MDA-MB-231 cells (Fig. 3C). As expected due to random cell seeding into the wells, we observed varying occupancy of the wells with cells ranging from 0 to 7 cells (Fig. 3D). Using the same approach as above, we collected and compared OCR and ECAR data of 1,297 single MDA-MB-231 and 1,321 single HME1 cells (Fig. 4). Compared with HME1 cells, the single cell OCR distribution of the MDA-MB-231 cells showed larger values, with some of the cells showing OCR as high as 4.8 fmoles/min (Fig. 4A). The number of MDA-MB-231 cells was lower in the range of 0.2–0.6 fmoles/min, but higher in the OCR range of 0.8–4.8 fmoles/min (Fig. 4A), as compared with HME1 cells. We found that the median OCR values of the two cell types (0.46 and 0.80 fmoles/min for HME1 and MDA-MB-231, respectively) were significantly different (p = 8.7·10^−19^, Kolmogorov-Smirnov (KS) two-sample test, Fig. 4B). Interestingly, we also found higher overall ECAR values of single MDA-MB-231 compared to HME1 cells (Fig. 4C). A comparison of the median ECAR values revealed a 4.5-fold larger ECAR in MDA-MB-231 cells than in HME1 cells (p = 9·10^−200^, KS test, Fig. 4D). Furthermore, the ECAR distributions of MDA-MB-231 cells were less heterogeneous in terms of coefficient of variation than HME1 (Supplementary Table 1). A scatterplot comparison of OCR and ECAR distributions (Fig. 4E) revealed an energy production phenotype with overall higher ECAR in MDA-MB-231 cells. Furthermore, the data showed the existence of a subpopulation of MDA-MB-231 cells with low OCR and ECAR (dashed oval in Fig. 4E) that overlap with a fraction of HME1 cells. These data illustrate the ability to measure OCR and ECAR simultaneously, allowing one to distinguish differences in energy production phenotypes between cell types with respect to OCR/ECAR ratio, provide details about phenotype variability on a cell-to-cell basis, and identify subpopulations of cells with differing energy production phenotypes by providing quantitative insight into the relative balance between the oxidative phosphorylation and glycolysis pathways.

### OCR and ECAR profiles in response to ETC inhibitors and uncouplers

**Figure 5:**
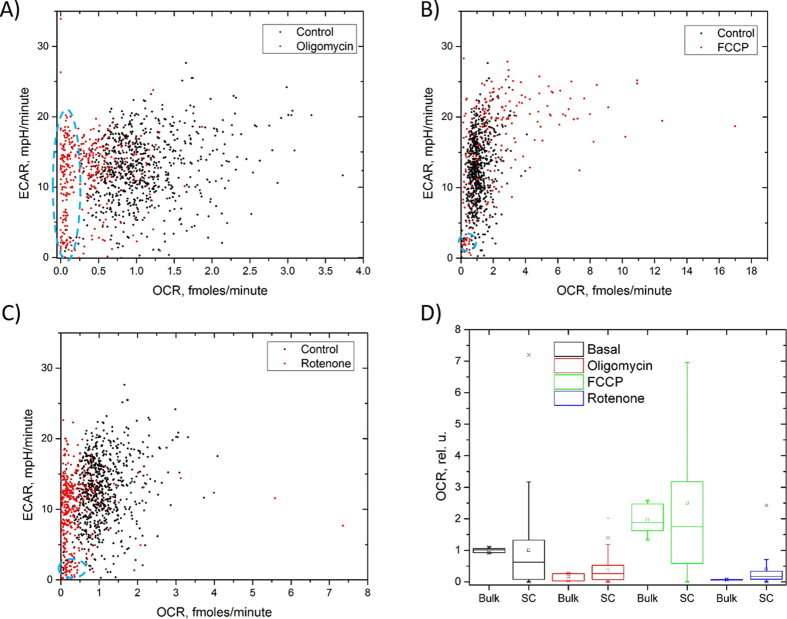
Validation of the approach using ETC inhibitors and an ion uncoupler with MBA-MD-231 cells. Single cell combined profiles of OCR and ECAR after treatment with oligomycin (complex V inhibitor) (**A**), FCCP (proton uncoupler) (**B**), and rotenone (complex I inhibitor) (**C**). All three treatments revealed highly variable responses of single cells and a subpopulation of cells with slow OCR (oligomycin) or slow both OCR and ECAR (FCCP, rotenone; blue dashed ovals). (**D**) A comparison of the relative changes in OCR measured with single cells with data obtained with bulk cell samples indicated both quantitatively and qualitatively similar behavior in response to the three perturbagens. Both data types were normalized against the basal respiration level for direct comparison.

To provide a quantitative validation of the Cellarium, we studied alterations in OCR and ECAR in the presence of ETC inhibitors and proton uncouplers. We measured OCR and ECAR of MDA-MB-231 cells in response to the addition of oligomycin, a complex V (ATP-synthase) inhibitor, to block ATP production via OXPHOS; carbonyl cyanide-4-(trifluoromethoxy)phenylhydrazone (FCCP), a proton uncoupler, to probe the maximal respiration rate, and rotenone, a complex I inhibitor. All three perturbagens resulted in characteristic changes that would be expected based on the mechanism of action. Namely, the data obtained with oligomycin showed a marked decrease in the average OCR value compared with the control cells, as evidenced by the almost complete absence of the cellular respiration in the majority of cells (Fig. 5A). Similarly, an about 2-fold increase in OCR was observed after treatment with FCCP (Fig. 5B). The addition of rotenone reduced the respiration rate to almost the same level as after the oligomycin treatment (Fig. 5C).

Next, we wanted to know how OCR and ECAR response to the two ECT inhibitors and the uncoupler would compare to that in bulk samples. To this end, we compared the single cell data with data obtained with bulk samples (1·10^4^–4·10^4^ cells/sample) of MDA-MB-231 cells. For bulk cell measurements we used a commercial platform (XFp, Agilent (formerly Seahorse), San Jose, CA) that has been utilized in numerous studies for mitochondrial function characterization^27^,^28^,^29^. For the purpose of comparison with other studies, we performed a standard mitochondrial respiration test implemented by the manufacturer. The protocol includes measurements of basal (no treatment) OCR and ECAR, and after administering oligomycin, FCCP, and rotenone. We found that changes in OCR were qualitatively and quantitatively similar between single cell and bulk cell data (Fig. 5D). Owing to the difficulty to determine accurately the number of cells in the bulk samples, we normalized the data to the corresponding basal OCR and ECAR values. The finding of almost identical average cellular behavior on the Cellarium platform in response to the ETC inhibitors and ion uncouplers indicates that population-level response to perturbagens measured at the single cell level closely resembles that of the bulk samples. However, our approach enables substantially deeper insight into cellular behavior by enabling data collection at the single cell level.

### Effects of cell-to-cell communication on bioenergy production

**Figure 6:**
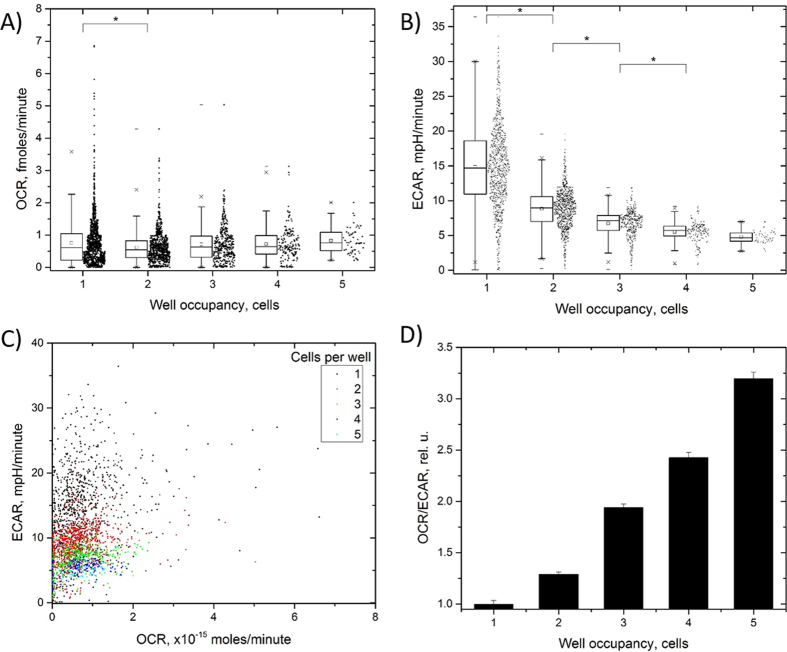
Effects of cell-to-cell communication on the bioenergy production phenotype of MDA-MB-231 cells. While OCR showed a slight, but statistically significant reduction between wells with one and two cells (**A**), the ECAR (**B**) profile exhibited a strong, statistically significant (p < 0.05, KS two-sample test) decrease with increasing number of cells per well. (**C**) Single cell distributions of OCR and ECAR as a function of number of cells per well. Marked shift towards lower ECAR with the increased cell number per well can be seen. The tightening of the distributions due to averaging over increasing number of cells can be observed. The relative change in OCR and ECAR (**D**) suggest a relative shift to a more mitochondrial bioenergy production profile (higher relative OCR).

The Cellarium microwell, with dimensions of 80 × 20 μm (Fig. 1) can accommodate up to 8–10 cells, depending on cell size. With random seeding, our approach provides a built-in capability to measure how the energy production profile is affected in the presence of cell-to-cell communication among varying numbers of cells. To demonstrate this capability we extracted OCR and ECAR data for cells that contained 2–5 cells per well and compared the values normalized to the number of cells in the well. We observed an overall decrease in the range of the OCR and ECAR values with increasing numbers of cells per well due to the averaging (Fig. 6A,B). Remarkably, we found that ECAR showed a strong downward trend (p < 0.05, KS two-sample test) with the increasing number of cells per well. On the contrary, OCR values exhibited a statistically significant difference between wells occupied with one and two cells, but were similar in wells with higher occupancy. Illustrating the averaging effect over multiple cells, distributions of OCR and ECAR became narrower with increasing number of cells per well (Fig. 6C). The overall OCR/ECAR distributions shifted to less acidic profile evidenced by a tilt towards the OCR axis. Furthermore, a comparison of the OCR/ECAR ratio (Fig. 6D) as a function of the number of cells per well revealed a strong upward trend and suggested an increase in the relative oxidative phosphorylation activity with increasing well occupancy.

## Discussion

The Cellarium approach and technology presented here for multiparameter energy production profiling in single cells enables characterization of transmembrane fluxes of oxygen and protons in real time. To the best of our knowledge, no other reported technology is capable of performing simultaneous characterization of OCR and ECAR of single cells with the throughput demonstrated in this study. In its current implementation, the technology enables the analysis of 200–300 individual cells per assay, while providing the built-in capability to study the response of multiple interacting cells per well for cell-cell communication studies. The comparison of HME1 and MDA-MB-231 cells revealed substantial variability in OCR and ECAR values at the single cell level. We found that while the majority of MDA-MB-231 cells exhibit marked differences from HME1 cells in terms of combined OCR and ECAR distributions, a subpopulation of MDA-MB-231 cells with both low OCR and low ECAR exists that are similar to the majority of HME1 cells. It is conceivable that these cells may also behave differently from the rest of the population in response to stress or treatment.

The data from wells containing more than one cell revealed a strong downward trend of ECAR with increasing number of cells. Furthermore, we observed a shift towards the mitochondrial production of ATP (higher OCR) relative to ECAR with increasing number of cells. These two findings suggest a role of intercellular interactions in energy production phenotype and demonstrates the capability of the approach to resolve details of such interaction. The effect of the number of interacting cells on OCR parallels our previous finding of altered OCR in Barrett’s esophageal epithelial cells that exhibited a significant increase in OCR in wells that were occupied with 3 cells^23^. The difference in OCR trends observed in this and our previous study suggests that the role of cell interaction in the bioenergy production phenotype is cell-type specific. This finding may bear direct relevance to the microenvironment of cancer cells, as the number of interacting cells within a tumor may also vary contributing to the observed metabolic heterogeneity^13^,^30^.

The validation assays performed with the ETC inhibitors and ion uncouplers (oligomycin, FCCP and rotenone) have revealed both qualitatively and quantitatively similar behavior of cells in our assays compared with bulk cell samples. This finding provides strong support for the non-perturbing nature of our approach in terms of cellular response and demonstrates the feasibility of stress-response studies using the platform. In all three cases our data revealed subpopulations of cells with slow OCR and ECAR in both control and treated cells. While further studies are necessary to reveal the functional role of these cells at the population and tissue level, it is possible that due to their dormant-like metabolic phenotype behavior these cells may play a role in drug response and cancer recurrence after treatment^31^,^32^.

Our data confirmed the findings of a previous study that demonstrated higher ECAR of MDA-MB-231 cells as compared with normal mammary epithelium (HME1) cells in bulk samples^26^. Moreover, the single cell OCR and ECAR distributions show differences between the two cell lines in terms of fractions of cells with low OCR and ECAR, indicating a substantially more complex behavior than one would assess based on the analysis of bulk cell samples alone.

The platform described here, has been engineered to be flexible, extensible, and allow higher levels of multiplexing. For example, more sensors can be incorporated for quantification of additional analytes, such as glucose, potassium, sodium etc., for more detailed studies of cellular metabolism. To achieve this, sensors with specific spectral properties may be designed for reliable separation of the emission signal from the different sensors. Furthermore, in addition to using just the sensor emission intensity as a readout, one can utilize time-resolved measurements of sensor emission decay times to calculate changes in analyte concentration^21^,^22^. Alternatively, the different sensors can be separated spatially inside the microwells using photo patterning or robotic sensor deposition, similar to the approaches utilized by our group^23^,^33^. The system throughput can be further increased by implementing experimental designs for running multiple assays in parallel. We have designed and tested an approach that enables running up to 5 assays in parallel thus bringing the overall throughput of the system to ~1,000–1,500 cells per assay. In addition to the assay parallelization, more advanced cell seeding approaches can be used to increase the number of wells containing single cells. For example, cells may be placed in the wells using inkjet printing^34^,^35^ or cell stenciling^36^ approaches with increased level of control over the cell number per well.

In summary, the Cellarium platform represents a powerful analytical tool enabling quantitative profiling of cellular bioenergy production in real time and at the single cell level. It provides the means to study the effect of cellular interactions on the bioenergy production profile at the few (2–5 cells) cell level. The approach is relatively simple in design and implementation, and can be expanded to accommodate more sensors for monitoring other analytes or products of metabolism or other cellular processes.

## Materials and Methods

### Sensor data acquisition

Fluorescent images of the oxygen sensor, pH sensor, and rhodamine containing film were acquired using an automated inverted epi fluorescence imaging platform (ImageXpress Micro XLS, Molecular Devices, Sunnyvale, CA). Fused silica wells with a volume of 64.5 pL were hexagonally packed into 12.8 × 12.8 mm arrays of up to 4,096 wells (Fig. 1). The data for the oxygen, pH and reference probes were acquired using excitation/emission filter sets of 390ex/655em, 475ex/536em and 542ex/593em, respectively.

### Image processing

The development of a high throughput single cell metabolic profiling platform required the development of an equally high throughput and robust image processing algorithm. Examination of platform performance and optimization of the platform protocol has become exceedingly facile through the development of near real-time metabolic data feedback accomplished using MATLAB (v. 2014b, Mathworks, Natick, MA) and the various toolboxes thereof. Automated image processing permits a strong degree of adaptability, the option to complete batch processing of hundreds of assays, and can implement quality control data filtering that would otherwise lead to misrepresentation of the metabolic profile.

Well lips within the microarray are readily apparent in the oxygen sensor fluorescent first time-point images within the drawdown component and the reference fluorescent images within the cell count component. These images are filtered for noise and corrected for non-uniform illumination or sensor concentration gradients and then converted to binary images such that the fraction of white within the image is equal to the fractional surface area of the well lips, ideally highlighting only the lips of the wells and blacking out everything else. Dark and light circular features within the logical images are then used as a template free method of detecting the wells through circular Hough transforms of the logical images (Supplementary Figure 4). The coordinates of each detected circle is filtered such that duplicate detections are weighted to a single detection according to their detection confidence metric. This redundant detection technique maximizes both the accuracy of the well coordinates and the quantity of detected wells. The coordinates are then filtered again for false positives by comparing the position of each coordinate to the coordinates nearby and discarding detections that do not agree with the geometry of the microarray. A cropped time-series subset of the assay wells are isolated and well detection is performed over the time-series to adjust the well coordinates of the assay for unexpected shifts (well tracking). Using a cropped subset of the images for well-tracking relies upon the fact that our assay is rigid (moves uniformly) and greatly reduces the processing time by subsampling the large data set for tracking analysis. The cell count well coordinates are first estimated using a non-reflective similarity transform of the well coordinates characteristic of the objective lens change from 4X to 10X magnification. These estimated cell count well coordinates are then compared to the detected well coordinates to synchronize the two assay components. The estimated cell count well coordinates are replaced with detected coordinates and an assay specific transformation is obtained for optimal alignment.

Implementing quality control on high throughput screening platforms is essential for maintaining data integrity. Well inspection is implemented on the Cellarium assays through an internal reference system which assumes that most of the data samples are valid. Segmentations of the first time-point rhodamine fluorescent image acquisition reveal two common rejection criteria during the well inspection subroutine of a custom image processing algorithm (Supplementary Figure 5). One of the wells has a microfabrication defect that resulted in incomplete well formation. This well cannot provide valid metabolic data as it would be free to exchange metabolites with the surrounding fluid volume (metabolic isolation is not fulfilled). Two of the wells are not fully sealed by the sensor or are on regions where sensor is only partially present, this also does not satisfy metabolic isolation and the lack of sensor may skew the extracted data from within the well (Supplementary Figure 5A). To detect defects the segmented images are divided by a reference image that is defined as the mean of all segmented images and then mean-normalized to one to account for any differences in bulk fluorescent intensity (sensor film heterogeneity). By this analysis the perfect well segmentation would contain the value of 1 for all pixels depicted as a saturated blue color (Supplementary Figure 5B). Pixels with values below one (unexpectedly darker) appear more dark blue, and pixels with values above one (unexpectedly brighter) appear more red. The coefficient of variation displayed as a percentage is then calculated for a quantitative measure of data integrity and outlier wells are discarded (Supplementary Figure 5C).

Three regions of data across 3 channels (O2 sensor, pH sensor, and reference) are extracted for each well for each time point within the drawdown images. The regions are specified by pixels whose centers fall within specified concentric circles (Supplementary Figure 6). Both mean and standard deviation values are acquired from these regions over time. The central region is to monitor the metabolism of cells contained within the microwell. The lip and outside regions are used as references for the surrounding environment to verify that intensity changes within the red circle are associated with the cell, and not associated with the surrounding environment.

Image segmentation is performed on the cell count images to obtain isolated crops of each well (Supplementary Figure 7A). The segmentation undergoes a well subtraction algorithm using the mean of all segmentation images which eliminates the well-associated artifacts from the image. This subtraction process is dynamic and catered to the average intensity of each well to obtain a clean cell isolation regardless of mean intensity. Image segments then undergo a variety of shading correction, image sharpening, noise reduction, and Laplacian of Gaussian based edge detection routines. Image processing parameters were optimized using a LaGrange multiplier minimization routine to adjust image processing and cell count decision routines. The objective function being minimized was the number of false cell counts as determined by the manual verification of 3,000 wells. Results from the Cellarium custom cell counting routine were compared to commercially available cell counting software and found to achieve the same level of accuracy, but took 50-fold less time to complete and integrated seamlessly with the drawdown data output. Cell counts for each well are manually verified using a custom approach taking approximately 15 minutes for every 1,000 wells.

### Sensor probe synthesis and sensor solution preparation

Polymerizable sensor probes—an oxygen probe (OS2), pH probe (S2), and an oxygen/pH insensitive reference probe (Rho-MA, Supplementary Figure 1A)—were prepared according to procedures published elsewhere^37^,^38^,^39^,^40^. The stock solutions of the oxygen sensor and pH sensor were prepared separately. The oxygen sensor stock solution was prepared by mixing 850 mg of 2-hydroxyethylmethacrylate (HEMA), 50 mg of acrylamide (AM), 50 mg of polyethylene glycol dimethacrylate (PEGDMA), 1 mg of Rho-MA, and 5 mg of OS2. The pH sensor stock solution was prepared by mixing 850 mg of HEMA, 50 mg of AM, 50 mg of PEGDMA, 1 mg of Rhod-MA, and 1 mg of S2. HEMA, PEGDMA and AM were obtained from Sigma-Aldrich (St. Louis, MO). Two hundred μL of each stock solution were mixed together followed by the addition of 6 mg azo-bis-isobutyronitrile. Then, 100 mg of 4-arm polyethylene glycol (PEG, MW = 5,000 g/mol, JenKem Technology, Allen, TX) was added and stirred for 20 minutes until PEG was completely dissolved. The solution was filtered through a 0.45 μm nylon membrane (VWR International, Radnor, PA) to yield a clear, dark pink solution.

### Sensor deposition

The sensor films with 3–4 μm thickness were created by sandwiching the sensor stock solution between a fused silica wafer that contained microfabricated shims of photoresists with a 5 μm height to provide a gap of desired size and a cover slip. To prepare the fused silica wafers, a photoresist SU8-2001 solution was made by diluting SU8-2005 (Microchem Corp., Westborough, MA) to a final solid concentration of 23% by using the SU8-2000 serial thinner (Microchem Corp.). The wafers were plasma treated in a plasma oven (Tegal Usher, CollabRx, San Francisco, CA) at 200 W for 10 minutes followed by a dehydration step at 160 °C for 10 minutes for improved binding of SU8 to the surface. Immediately following dehydration the SU8-2001 solution was applied to the wafer and spin coated at 3,000 rpm for 45 seconds. The coated wafers were pre-baked at 95 °C for 5 minutes on a hot plate and then exposed at 80 mJ of UV light using a mask aligner (OAI 808 aligner, OAI LLC, San Jose, CA) and a UV filter (PL-360LP, Omega Optical Inc., Brattleboro, VT). The exposed wafers were post-baked at 95 °C on a hot plate for 5 minutes and developed in a SU8 developer (Microchem Corp.) for 1 minute followed by a hard bake step at 150 °C for 30 minutes. The wafers were plasma treated together with the cover glass slides using a plasma cleaner (Harrick PDC-001, Harrick Plasma, Ithaca, NY) at 30 W power for 10 minutes to activate the glass surface for a silanization step. The wafers were vapor-phase silanized by placing them in a glass dessicator along with 200 μL of (3-acryloxypropyl) trimethoxysilane (4369-14-6, Gelest, Morrisville, PA) in an open 1.5 mL Eppendorf tube, and then evacuating the air using a house vacuum for 30 seconds until an absolute pressure of less than 20 mmHg in the desiccator was achieved. The wafers were kept in the desiccator for 4 hours. The cover slips were silanized using the same procedure, but using (1H,1H,2H,2H-perfluorooctyl) trichlorosilane (Gelest) to prevent the sensor from adhering to the surface. The sensor was cured (polymerized) in the sandwich structure at 80 °C under 100% nitrogen for 90 minutes. The cover slip was removed from the polymerized membrane surface after curing. The polymerized sensor films on the substrate were washed three times with methanol to remove any remaining sensor monomers and any residual solvents.

### Microwell chip fabrication

The microwell arrays were fabricated using photolithography as described elsewhere^41^,^42^. Briefly, first a thin layer of amorphous silicon was uniformly deposited on the surface of an RCA-cleaned fused silica wafer. Then the wafer was spin-coated with AZ 4330 photoresist at 3,000 rpm, and UV exposed at a dose of 150 mJ/cm^2^. The photoresist was patterned with lipped microwell arrays, and the patterns were transferred to the amorphous silicon layer by a dry reactive-ion etching (RIE) process. The patterned amorphous silicon layer worked as a masking layer during HF wet etching to form the microwell arrays with the height of 15 μm. After HF wet etching, the amorphous silicon left on the fused silica was completely removed by a second RIE step.

### Microwell substrate preparation

The microwell substrates were sonicated in a 1% detergent solution (micro-90, VWR, Radnor, PA) for 30 minutes to remove glass shards or particles from the surface followed by a cascade rinse with deionized ultrapure water to remove residual detergent. The substrates were sprayed with 70% ethanol and allowed to air-dry. Next, the substrates were placed in a 35 × 10 mm polystyrene Petri dish containing 2 mL of the appropriate cell culture medium and kept under normal cell culture conditions for 24 hours to remove any air trapped in the wells. The cell culture medium was removed the next day and replaced with 2 mL of fresh culture medium containing 160,000–250,000 cells. Optimal cell number was determined empirically for each cell line (see separate section for cell culture conditions). The substrates were incubated under normal cell culture conditions for 24 hours prior to experiments, which allowed the cells to adhere to the surface of the microwell substrate and equilibrate. The next day, the microwell substrates and cells were imaged with 5x and 10x objectives (Evos XL Core microscope, Thermo-Fisher Scientific, Carlsbad, CA) to examine cell morphology. Prior to the assay, the 2 mL of media was removed and replaced with 2 mL of assay medium comprised of 0.8 mM MgSO_4_ 1.8 mM CaCl_2_, 143 mM NaCl, 5.4 mM KCl, 0.91 mM NaH_2_PO_4_, 4.5 mM glucose, and 1 mM Hoechst 33342 nuclear stain for cell counting. The cells were incubated in assay medium for 15 minutes before the experiment.

### Sensor preparation for measurements

The sensors were prepared for assays by soaking in 100% methanol for 15 minutes to remove potential un-cured sensor material. They were then cascade-rinsed with deionized water to remove methanol. Next, the sensors were sprayed with 70% ethanol and allowed to air-dry. Immediately prior to assay, a sensor was soaked in the final assay medium for at least 30 minutes to equilibrate the sensor with assay medium, which was found to result in a more stable response during assay.

### Oxygen Sensor Calibration

The oxygen sensor phosphorescence intensity changes as a function of dissolved oxygen concentration due to dynamic quenching of the triplet energy state by oxygen (Supplementary Figure 1B,C) and can be described using the Stern-Volmer relationship:


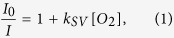


where I is the sensor emission intensity at dissolved oxygen concentration [O_2_], I_0_ is the sensor emission intensity at 0% oxygen and k_SV_ is the Stern-Volmer constant of dynamic quenching efficiency. The sensor response was calibrated using a 2-point calibration in assay medium with dissolved oxygen concentration ranging from air-saturated (first calibration point) to 0% (last calibration point) dissolved oxygen conditions at 37 °C. To achieve the 0% dissolved oxygen condition, the assay medium was purged with 100% nitrogen for 10–20 minutes. The absolute concentrations of dissolved oxygen for the two calibration points were confirmed using a calibrated Clark electrode. The I_0_/I_air_ ratio in the assay medium at 36 °C and 20 kPa oxygen partial pressure was 2.7–3.0 and varied slightly from sensor to sensor. The k_SV_ was determined by linear regression from


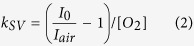


To improve oxygen measurement accuracy, we performed a local, well-by-well calibration of the sensor response by inference using assay results. Using the same two-point approach, the local sensor calibration was inferred from the sensor emission intensities at the point of sealing/assembly (air-saturated condition) and at the point where the well is fully drawn down (0% dissolved oxygen). An average value of k_SV_ was calculated for the assay by averaging k_SV_ over all wells that completed assay. The average k_SV_ was then applied equally to all wells, including wells that did not draw down completely such as wells with no cells. With this approach, either the final value or the initial value of oxygen could be used as the calibration reference.Concentration for all cases was found from the following equation:


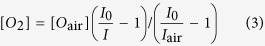


where [O_air_] is dissolved oxygen concentration under air-saturated conditions.

### pH Sensor Calibration

The pH sensor intensity as a function of pH can be modeled with the sigmoid function:


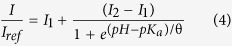


where I_1_ and I_2_ are the maximum and minimum normalized intensity of the sensor dynamic range, respectively, pKa is the acid dissociation constant, θ is sensitivity, and I_ref_ is the sensor intensity in the standard assay medium. The sensor was calibrated using a Britton-Robinson buffer with varying pH values in the range of pH 3–9 (Supplementary Figure 1D). Fluorescence images of the sensor were acquired at 5–10 min after each change of the buffer to a different pH to allow sensor equilibration. The fluorescent intensities were normalized to the pH of the assay medium. Data collected across 11 sensor samples were compiled and fit to equation (4). A typical curve fit is shown in Supplementary Figure 1E. We observed a typical change in pH during assays in a range of 6.4–7.4. The response is close to linear in this range.

### OCR and ECAR calculations

OCR and ECAR were calculated by applying linear regression to oxygen concentration and pH data, respectively. The estimated average confidence interval on the slope was calculated to be ±14%. ECAR was calculated from the slope of the pH curve and had units of pH/minute. OCR is calculated from the slope of the OC curve and has units of fmoles/min. Because of the random seeding process, a proportion of wells had no cells. We used the empty wells as negative controls to ensure the absence of sensor response during assays. However, a slight (5–10%) increase in emission intensity was observed in the oxygen sensor associated with sensor consumption of oxygen. By calculating the effective OCR of empty wells, we have estimated the associated error in OCR for wells with cells.

### Sealing microwell array and data collection

A custom fixture was designed which aligns the microwell substrate to the sensor substrate, contains the assay medium, maintains consistent temperature, and, when closed, produces balanced and sufficient force for hermetic sealing of the wells (Supplementary Figure 2). The microwell substrates containing cells were placed in the lower part (base block) of the fixture. Assay medium (100 μL) was added on top of the substrate. The sensor was placed on top of a black adhesive foil (Spectral Black Adhesive Foil, Edmund Optics, Barrington, NJ) pre-cleaned with 70% ethanol that was pre-attached to the compression plate and wetted with 60–80 μL of assay medium prior to assay. The light absorbing foil material was used to decrease non-specific reflected light. Then the lower and upper fixtures were closed and put into the ImageXpress Micro XLS platform for imaging. The sensor was sequentially imaged in three spectral windows: 390ex/655em (oxygen sensor), 475ex/536em (pH sensor), and 542ex/593em (reference probe).

### Verifying seal of microwells (seal test)

At the end of the assay, 100% oxygen was flowed through the fixture at 20 cm^3^/minute for approximately 40 minutes using a flow controller (Alicat Scientific, Tucson, AZ) while imaging continued. The flow rate was selected to purge air from the fixture internal volume, but without causing the liquid between the substrates to dry out. The oxygen sensor intensity in sealed wells on the array did not show measurable changes, whereas the intensity inside partially sealed wells decreased with time. The oxygen purge was confirmed by the marked reduction in emission intensity of the sensor areas outside microwells.

### Staining of cell nuclei for counting

Because of random cell seeding into the wells, counting of cells in each microwell was necessary to determine the number of cells per well (Fig. 2A,B, Supplementary Figure 7A). To this end, the cell nuclei were stained with the Hoechst stain for nuclear DNA and the entire microwell array was imaged with a 10x objective lens in fluorescence mode using 390ex/520em and 542ex/593em spectral windows to visualize the cells and determine the location of the wells, respectively.

### OCR and ECAR in bulk samples

The OCR and ECAR measurements in bulk cell samples were performed using the Seahorse XFp metabolic flux analyzer (Agilent, San Jose, CA). The day before the assay, 10,000–35,000 cells/well were seeded into XFp cell plates coated with poly-d lysine. The cells were plated at ~80% confluence in 75 μL of the appropriate medium for the cell type. The plates were left in the cell culture hood for 30–45 minutes to allow cell attachment. Then, an additional 120 μL of culture medium was added to all of the wells and the plate was covered and incubated overnight at 37 °C with 5% CO_2_. Prior to experiments the next day, the cell medium was replaced with an assay medium consisting of 5 mM glucose, 2 mM glutamine, and 1 mM pyruvate in the Seahorse base medium. The plates were placed in a non-CO_2_ incubator for 40 minutes prior to the start of the assay. The assays were run according to the manufacturer’s mitochondrial stress test. The following perturbagen concentrations (μM) were used: 1 (oligomycin), 1 (FCCP), 0.5 (rotenone).

## Additional Information

**How to cite this article**: Kelbauskas, L. *et al*. A platform for high-throughput bioenergy production phenotype characterization in single cells. *Sci. Rep.* **7**, 45399; doi: 10.1038/srep45399 (2017).

**Publisher's note:** Springer Nature remains neutral with regard to jurisdictional claims in published maps and institutional affiliations.

## Supplementary Material

Supplementary Figures
