# Supplementary material for: A platform for high-throughput bioenergy production phenotype characterization in single cells
Source: Sci Rep. 2017 Mar 28;7:45399. doi: 10.1038/srep45399 (PMC5368665; doi:10.1038/srep45399)
Supplement: Supplementary Figures [file srep45399-s1.docx]

**A platform for high-throughput bioenergy production phenotype characterization in single cells**

Laimonas Kelbauskas, Honor Glenn, Clifford Anderson, Jacob Messner, Kristen Lee, Ganquan Song, Jeff Houkal, Fengyu Su, Liqiang Zhang, Yanqing Tian, Hong Wang, Kimberly Bussey, Roger Johnson, and Deirdre R. Meldrum*

Center for Biosignatures Discovery Automation, The Biodesign Institute, Arizona State University, 1001 S. McAllister Ave., Tempe, AZ 85287

*Corresponding author (e-mail: [deirdre.meldrum@asu.edu](mailto:deirdre.meldrum@asu.edu))

# Supplementary Figures


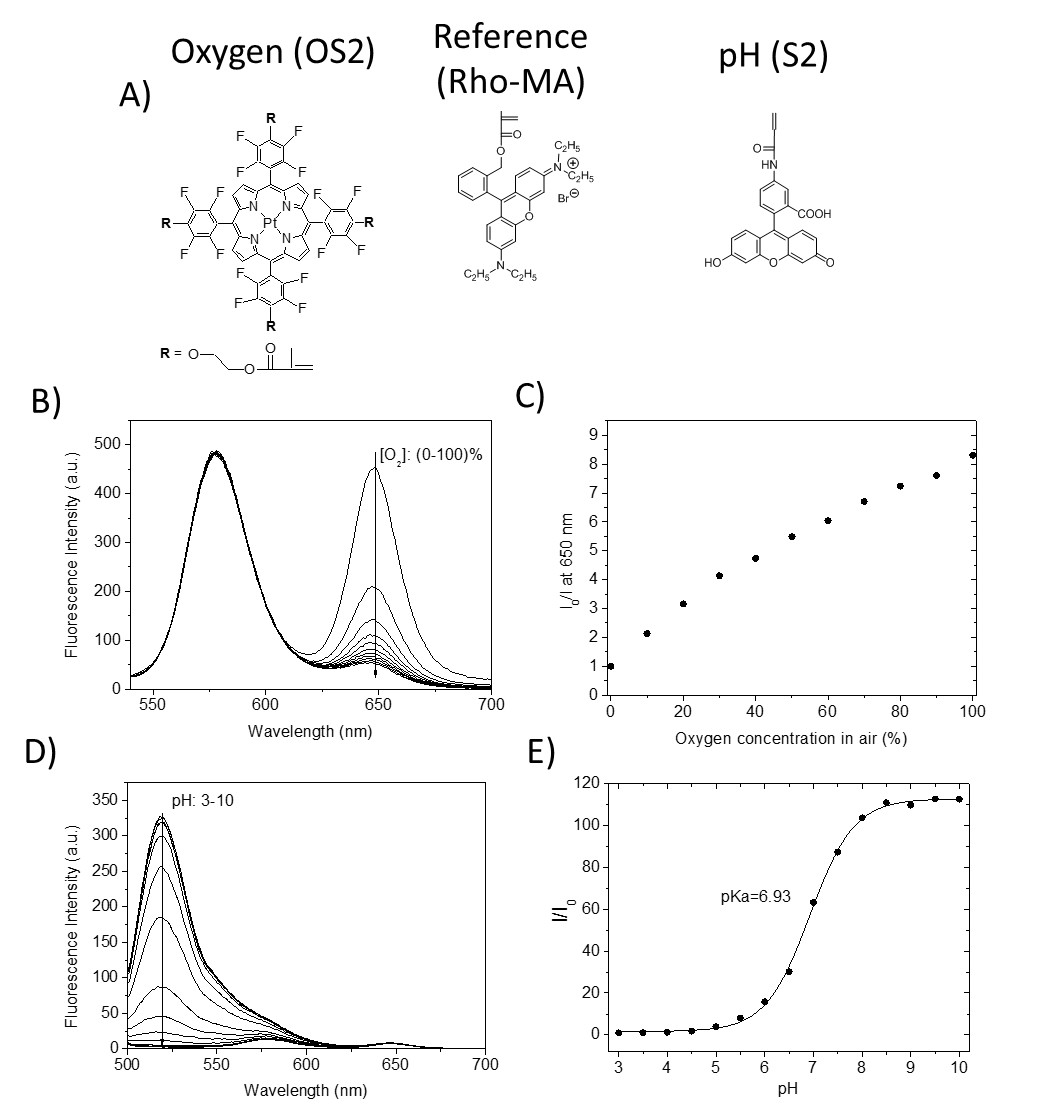


**Supplementary Figure 1.** Optical pH and oxygen sensor with a built-in reference probe. A) Molecular structure of the sensors and the reference probe; B) Emission spectrum of the sensor in response to different levels of dissolved oxygen. The emission peak centered at 580 nm is signal from the reference probe and does not respond to alterations in oxygen concentration; C) Inverted relative sensor response to changes in dissolved oxygen concentration in aqueous solution; D) Changes in sensor emission spectrum in response to varying pH. The two smaller emission peaks centered at 580 nm and 650 nm are reference and oxygen sensor emission, correspondingly, and show no response to pH changes; E) Sigmoidal relative response of the sensor (solid curve represents a theoretical fit) to changes in pH showing excellent sensitivity in the physiological range with pKa of 6.92.


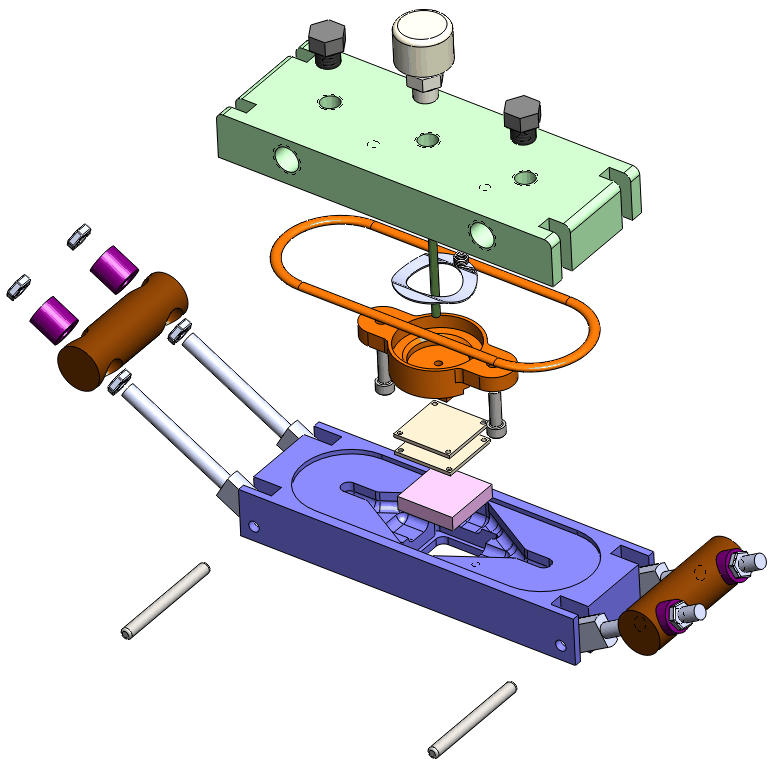


Cap block

Fluid path access caps blockFluid

Spring

(compression force)

Chamber seal

Compression plate

Window

Microwell array

Sensor chip (lid)

Pivot rods

Handle

Base block

**Supplementary Figure 2.** A 3D rendering of the fixture design for hermetic seal production in microwells. The lid and microwell array are brought together inside the base block and sealed by lowering down the cap block that contains a spring-loaded compression plate to produce a controlled amount of pressure for sealing. The fixture is secured with the spring-loaded handles during the assay. The glass window serves as an optically compatible support for imaging. The ports in the cap block allow for gas to be purged on top of the fluid inside the sealed fixture for calibration and seal testing purposes.

|  |
| --- |

**Supplementary figure 3.** Flow chart depicting the data acquisition and analysis pipeline. Three different series of images are taken during a typical assay: 1. Sensor emission images for OCR and ECAR calculations (time series); 2) a high resolution fluorescence image of stained cell nuclei of the entire microwell array for cell counting; 3) oxygen sensor images for hermetic sealing validation (time series). All assay images are stored in a database prior to processing. A two-pronged image processing is used, consisting of sensor data extraction from the sensor time series and counting the number of cells per well from the cell nuclei image. Hermetic seal images are used for determining which wells were completely sealed during the assay.


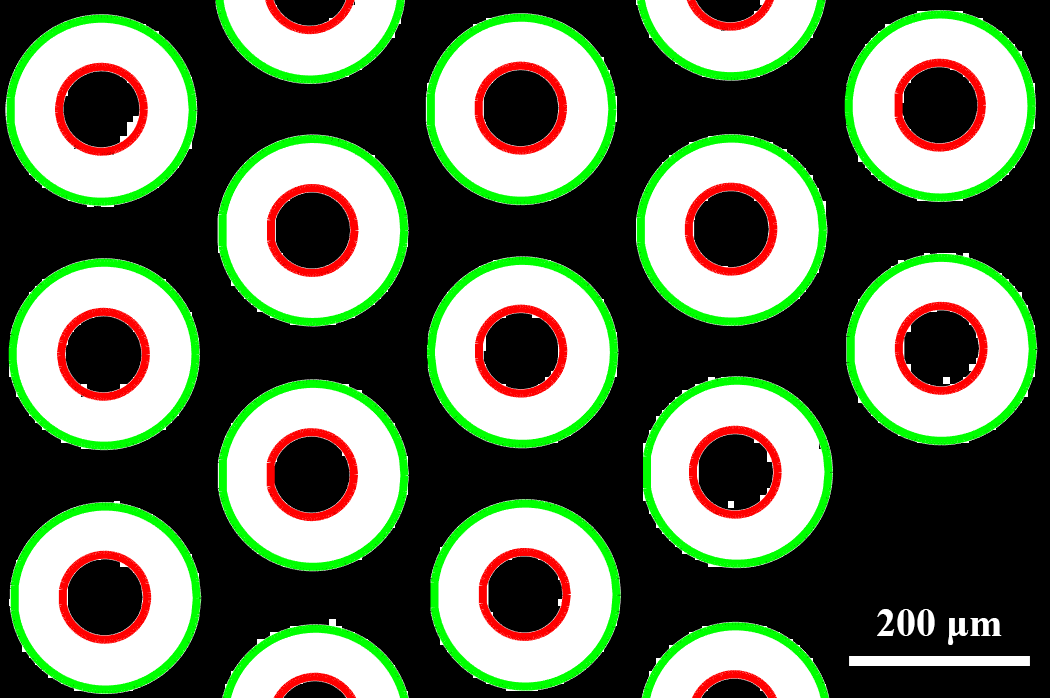


**Supplementary Figure 4.** Well detection based on the circular Hough transform of a binary assay image. The white circles represent microwells after intensity-based thresholding of fluorescence images. Red = dark well detection, Green = bright well detection. Binary images were generated from images of the reference fluorophore channel by applying an intensity threshold that was empirically determined to accentuate the well lips.


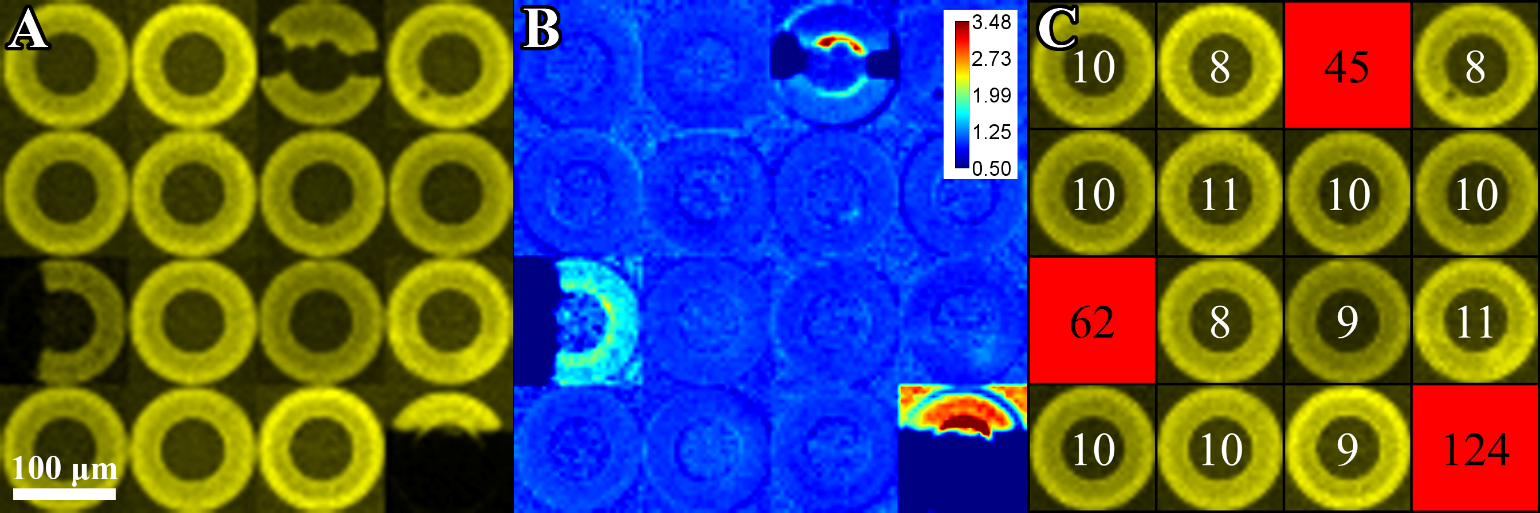


**Supplementary Figure 5.** Well damage inspection and rejection outline for a typical assay. (A) Montage of raw rhodamine channel image segments illustrating heterogeneous data samples; (B) Rhodamine image segments divided by a representative template image segment and normalized to 1; (C) Coefficients of variation (%) derived from images in B, wells covered by red box were discarded from analysis through outlier criteria.


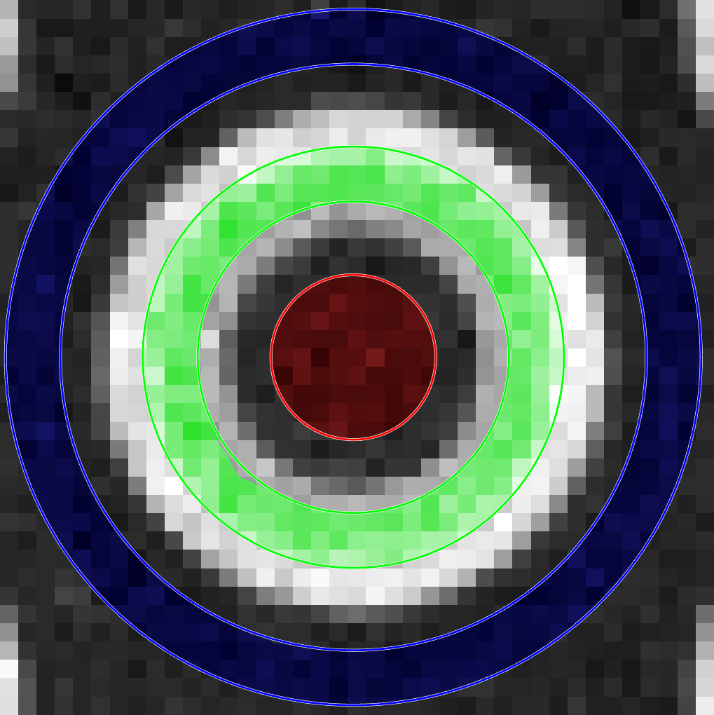


**Supplementary Figure 6.** Data extraction regions example from a microwell during an assay. Extracted pixel values are highlighted in red (well interior), green (well lip), and blue (well exterior). Sensor intensity values from the different regions of the well can be used for calculations of the gradients of oxygen or pH for leak rate determination. Mean and standard deviation values are reported from each well for each fluorescent channel across a number of time points.


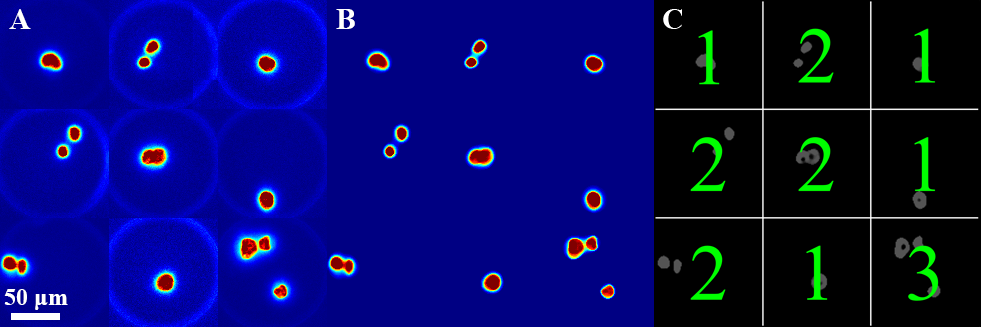


**Supplementary Figure 7.** Cell count processing overview. (A) Montage of Hoechst 33342 (nuclear DNA stain) well images with red features denoting highly fluorescent nuclei. (B) Images from A after subtracting the wells. (C) Numbers of nuclei (cells) per well as determined using a custom algorithm with high accuracy.

**Supplementary Table**1. Descriptive OCR and ECAR statistics of the two studied cell lines.

| **Cell line** | **N** | **Parameter^§^** | **Mean** | **SD** | **CV** | **Median** | **Range** |
| --- | --- | --- | --- | --- | --- | --- | --- |
| HME1 | 1,321 | **OCR** | **0.46** | **0.45** | **0.98** | **0.34** | **2.31** |
|  |  | ECAR | 3.22 | 2.75 | 0.86 | 2.50 | 20.04 |
| MDA-MB-231 | 1,297 | **OCR** | **0.80** | **0.76** | **0.94** | **0.63** | **6.61** |
|  |  | ECAR | 14.41 | 6.27 | 0.44 | 14.46 | 36.41 |

^§^Units: OCR – fmoles/minute, ECAR – mpH/minute; SD – standard deviation, CV – coefficient of variation.
